# Teaching a single manual therapy technique at a time reduces cognitive load in physiotherapy students: a randomized controlled educational study

**DOI:** 10.1186/s12909-025-08083-w

**Published:** 2025-10-15

**Authors:** René Gärtner, Harm Peters, Ylva Holzhausen

**Affiliations:** 1 Ludwig Fresenius School of Physiotherapy, Berlin, Germany; 2 Dieter Scheffner Centre for Medical Education and Educational Research, Dean’s Office of Study Affairs, Charité - Universitätsmedizin Berlin, Campus Charité Mitte, Charitéplatz 1, Berlin, 10117 Germany

**Keywords:** Cognitive load theory, Manual therapy, Physiotherapy, Instructional Design, Medical Education

## Abstract

**Background:**

Manual therapy is a fundamental component of physiotherapy education, requiring students to develop complex procedural skills through structured instructional methods. Sweller’s cognitive load theory can provide a framework in manual therapy education on how teaching design affects learning efficiency, especially in the acquisition of procedural skills. This study examines the impact of different teaching approaches on students’ cognitive load in manual therapy education.

**Methods:**

A randomized controlled educational study was conducted with 48 physiotherapy students from two cohorts in spring 2022 and 2023. Participants were randomly assigned to an individual practice group (learning one technique at a time) or a series practice group (learning 3–4 techniques simultaneously). A questionnaire assessed global cognitive load as the primary outcome and intrinsic, extraneous, and germane cognitive load as secondary outcomes.

**Results:**

477 questionnaires were analysed. Global cognitive load was significantly lower in the individual group than in the series practice group (MD = -0.55, 95% CI -0.87 to -0.22). Compared to the series practice group, the individual practice group had lower intrinsic cognitive load (MD = -0.29, 95% CI -0.40 to -0.18), slightly lower extraneous cognitive load (MD = -0.07, 95% CI -0.13 to -0.01), and reduced germane cognitive load (MD = -0.29, 95% CI -0.43 to -0.17).

**Conclusion:**

Teaching manual therapy one technique at a time may reduce cognitive load, potentially enhancing student learning and performance. This approach underscores the value of applying cognitive load theory in instructional design for physiotherapy education, offering practical benefits for teaching strategies.

**Supplementary Information:**

The online version contains supplementary material available at 10.1186/s12909-025-08083-w.

## Background

Manual therapy is an essential aspect of physiotherapy. It comprises a variety of hands-on techniques aimed at mitigating pain, enhancing mobility, and improving overall function. For physiotherapy students, the proficient acquisition of manual therapy techniques is central to their preparation for future professional practice. However, few studies have explored the factors that influence the learning process and the effectiveness of different educational strategies [1]. In other settings, students’ cognitive load in a teaching session has been associated with learning outcomes [2–5]. Thus, it would be interesting to identify ways to reduce students’ cognitive load in manual therapy teaching sessions [6]. This study in particular aimed to explore the effects of different teaching techniques in manual therapy on students’ cognitive load.

As a professional practice, manual therapy involves the use of skilled hand movements to manipulate muscles, joints, and soft tissues [7]. A systematic educational approach that combines theoretical knowledge with practical skill development is needed for the instruction of manual therapy techniques [8]. which are generally taught through a structured curriculum with a blend of lectures or seminars, demonstrations, supervised hands-on practice, and continuous feedback. A common educational approach to facilitate comprehension and skill acquisition is teaching and practising small series of manual therapy techniques in small groups [9]. Traditionally, these sessions involve a sequence of live demonstrations, during which the teacher supervises and provides individual feedback on students’ performance [10].

Although the teaching of manual therapy is an essential part of physiotherapy curricula, few studies have examined this field, and little is known about the best way to structure teaching sessions [10, 11]. A randomized study by Rossettini and colleagues of third-year physiotherapy students compared the traditional teaching method of “see one, do one” with Peyton’s four-step approach” for the acquisition of manual therapy techniques, i.e., passive right mobilization of the cervical spine at the C1-C2 level [1]. This study revealed that when Peyton’s four-step approach was used, the quality of skill performance was better both immediately and one month following the teaching session when a preestablished skills checklist was used. This study has shown that reconsidering the standard structure of teaching sessions may be beneficial [12].

To date, the theory of cognitive load has not been utilized to improve the learning of manual therapy techniques. While cognitive load is often discussed in the context of instructional efficiency, it is important to note that cognitive load is not inherently detrimental. Rather, it is a natural component of learning that engages working memory resources for schema construction and automation [13]. Indeed, certain motor learning paradigms suggest that increased cognitive load can enhance long-term retention and transfer, such as in dual-task training or implicit learning approaches [14]. Thus, understanding how teaching methods influence the type and magnitude of cognitive load is critical for optimising learning outcomes. Cognitive load theory, which was introduced by John Sweller, is rooted in cognitive psychology and provides a framework for understanding how the human cognitive architecture interacts with information presented during learning [15, 16]. This theory posits that working memory has a limited capacity and that learning is impaired when the cognitive load imposed by instructional materials exceeds this capacity [17]. Cognitive load can be categorized into three types: intrinsic, extraneous, and germane [18]. Intrinsic cognitive load is related to the inherent complexity of the task itself [19]. Extraneous cognitive load is related to the way in which information is presented to the learner, and germane cognitive load refers to the working memory resources that are devoted to processing, constructing and automating schemas—mental constructs that help to understand and perform tasks more efficiently. Thus, cognitive load theory provides a theoretical framework for understanding how information processing demands might affect learning outcomes [19].

The effects of different teaching methods on students’ cognitive load have been described in other settings [5, 20–24]. The cognitive load associated with the acquisition of manual therapy techniques may be considerable, as these techniques are often complex and highly procedural [9]. Specifically, physiotherapy students need to integrate cognitive, psychomotor, and affective skills. Moreover, they need to remember, interpret, and execute multiple steps and adjust to feedback during practical applications. If the cognitive load imposed by a learning task is too high, it might interfere with the learner’s ability to effectively acquire and retain the skill [13, 25]. Thus, cognitive load can be measured to better understand the interplay between cognitive load and the acquisition of manual therapy techniques and to modify the way they are taught to physiotherapy students.

The aim of this randomized study was to compare the cognitive load of two groups of physiotherapy students learning the same manual therapy techniques. The primary outcome is the effect on the global cognitive load, and the secondary outcome is the effect on the intrinsic, extrinsic and germane cognitive loads. The following two research questions were formulated:

1. Does individualized teaching and practice of manual therapy techniques reduce physiotherapy students’ global cognitive load compared to teaching and practicing multiple techniques simultaneously?
2. How do variations in teaching techniques influence intrinsic, extrinsic, and germane cognitive load components?

It was hypothesized that students’ overall global cognitive load would decrease when only one manual therapy technique is demonstrated at a time by the instructor and subsequently practiced by the students.

## Methods

### Centres, participants, and therapists

In Germany, physiotherapy training is not university-based but is instead structured as a continuous dual training program lasting three years, with admission requiring at least a tenth-grade diploma. This study was conducted at the Ludwig Fresenius Physiotherapy School in Berlin, Germany. Its physiotherapy study program comprises a total of 100 hours of manual therapy instruction in year 2 of study, including practical and oral assessments. The focus of the manual therapy course is joint treatment, including “mobilizing joints with limited range of motion.” In the preceding curriculum, physiotherapy students already had skills training in the application of general practical physiotherapy techniques.

This study involved the 2022 and 2023 cohorts of second-year physiotherapy students, with a total of 11 manual therapy training sessions for each cohort. Each teaching session was attended by 22–24 students. An overview of the manual therapy techniques taught is provided in the Appendice. The sessions followed the sequence of starting with upper extremity techniques, proceeding to the lower extremity, and ending with spinal techniques. Instructions for both the upper and lower extremity techniques, as well as the spinal techniques, followed a proximal-to-distal progression (upper extremity: shoulder girdle → shoulder joint → elbow joint → wrist joint → finger joints; lower extremity: hip joint → knee joint → ankle joints → toe joints; spine: cervical spine → cervicothoracic junction → thoracic spine → lumbar spine → iliosacral joint).

The author RG taught all the manual therapy sessions. To avoid bias, RG followed a standardized teaching script for manual therapy techniques in all sessions, which was made available for download to all students, regardless of whether they were in the intervention or control group.

### Study design and intervention

This randomized controlled educational study was embedded within a manual therapy training course.

At the beginning of each of the 11 manual therapy session, the students were randomly assigned to one of the following groups via covered index cards: (1) In the series practice group, the students watched a demonstration of a series of 3–4 new manual therapy techniques by the teacher. The students then practiced a series of manual therapy techniques in pairs of two students at their treatment benches alternating between the roles of patient and therapist. (2) In the individual practice group, the students watched a demonstration of one new manual therapy technique by the teacher, after which they went to their treatment benches to practice the new technique in pairs, also alternating between the roles of patient and therapist. The next new manual therapy technique was then demonstrated to the students, followed by another period of practice until all 3–4 manual therapy techniques were covered. The number of manual therapy techniques and their order were identical in the two study groups. During the practice period, the teacher visited each training table to observe the execution of the technique and to provide feedback.

After the training, the students reported their cognitive load during the teaching and practice of the manual therapy techniques via a Likert-scale-based questionnaire. Study participation was voluntary and required written informed consent.

Students could be allocated to both study groups over the course of all sessions. However, due to practical considerations and prior experience with student surveys, we did not assign participant IDs and completed questionnaires could not be linked to individual participants. The students were instructed not to discuss group allocation or techniques outside of class to minimise contamination.

### Questionnaire development and data collection

The authors developed the questionnaire in an iterative process on the basis of the literature on measuring the cognitive load during the learning of procedural skills. The items of the questionnaire used in this study are shown in Table 1.

To capture the global cognitive load, the mental-effort rating scale by Paas was used [26]. The students were asked to indicate their mental effort when carrying out the manual therapy techniques on a 9-point Likert scale (1 = very, very low; 2 = very low; 3 = low; 4 = rather low; 5 = neither low nor high; 6 = rather high; 7 = high, 8 = very high; 9 = very, very high).

**Table 1:** Items of the administered questionnaire

Categorie	Items
Global cognitive load	My mental effort when carrying out the manual therapy treatment technique was
nine-point scale:	
very very low –	
very very high	
Intrinsic cognitive load four-point scale: • disagree • tend to disagree • tend to agree • agree	I1. It was difficult to carry out the manual therapy treatment technique in the specified sequence and manner shown.
	I2. It was difficult to identify normal anatomy and/or landmarks.
	I3. It was difficult to keep track of or remember all the findings.
	I4. Overall, this manual therapy treatment technique was difficult and/or complex.
Extraneous cognitive load four-point scale: • disagree • tend to disagree • tend to agree • agree	E1. My supervisor’s instructions were unclear.
	E2. The manner in which my supervisor provided instructions or teaching was ineffective for my learning.
	E3. I felt distracted by other people present in the room.
	E4. I felt distracted by the environment (i.e., background noise, the layout of the room).
	E5. I felt distracted by things on my mind unrelated to this treatment.
Germane cognitive load four-point scale: • disagree • tend to disagree • tend to agree • agree	G1. I invested substantial mental effort in learning how to carry out the new manual therapy treatment technique.
	G2. I invested substantial mental effort in identifying or understanding the anatomy of the particular structure being treated.
	G3. Overall, I invested substantial mental effort in learning during the manual therapy treatment technique.

To capture the subdimension of cognitive load, it was built upon the Cognitive Load Inventory for Colonoscopy (CLIC) questionnaire, which was developed and validated by Sewell and colleagues for colonoscopy training [27]. The CLIC questionnaire was adapted by the authors to the context of manual therapy technique training. In December 2021, the questionnaire was pilot tested on a group of second-year physiotherapy students to check for clarity of the item wording and the application in the context of their training in manual therapy techniques. Some minor adjustments were made to the questionnaire on the basis of their feedback. A 4-point Likert scale (1 = disagree − 4 = agree) was chosen for the students’ ratings to avoid a tendency in the rating towards the middle.

### Outcome measures

The primary outcome was the effect of the different teaching modes on the global cognitive load during the performance of the manual therapy techniques, which was assessed on the basis of student ratings. The secondary outcome was the effect of the different teaching modes on the categories of cognitive load, such as intrinsic, extraneous and germane loads, during the performance of the manual therapy techniques, which was also assessed on the basis of student ratings.

### Data analysis

The data were analysed via the open-source software JASP 0.18 (Apple Silicon). The responses for each set of items in the categories of cognitive load were averaged to calculate the total intrinsic, extraneous and germane loads. The internal consistency of the individual items in a cognitive load subdimension was tested via Cronbach’s alpha calculation. Descriptive results are expressed as mean (M) and standard deviation (SD). To compare the means of the two study groups, the mean group differences (MDs) with 95% confidence intervals (CIs) were calculated.

A priori power analysis was conducted using JASP 0.18 (Apple Silicon) to determine the required sample size for detecting the expected effect. Assuming a Cohen’s d of 0.60, an Cronbach´s α level of 0.05 (two-tailed), and a desired power of 0.80, the analysis indicated that 24 participants per group were required.

## Results

In year 2022 and 2023, a total of 50 students was enrolled in the manual therapy course and 48 agreed to take part in the study and provided informed consent (22 students in year 2022 and 26 students in year 2023). 23 (48%) of the participants were female, and 25 (52%) were male. The mean age at study entry was 23.9 years (SD 5.6 years).

**Fig. 1:**
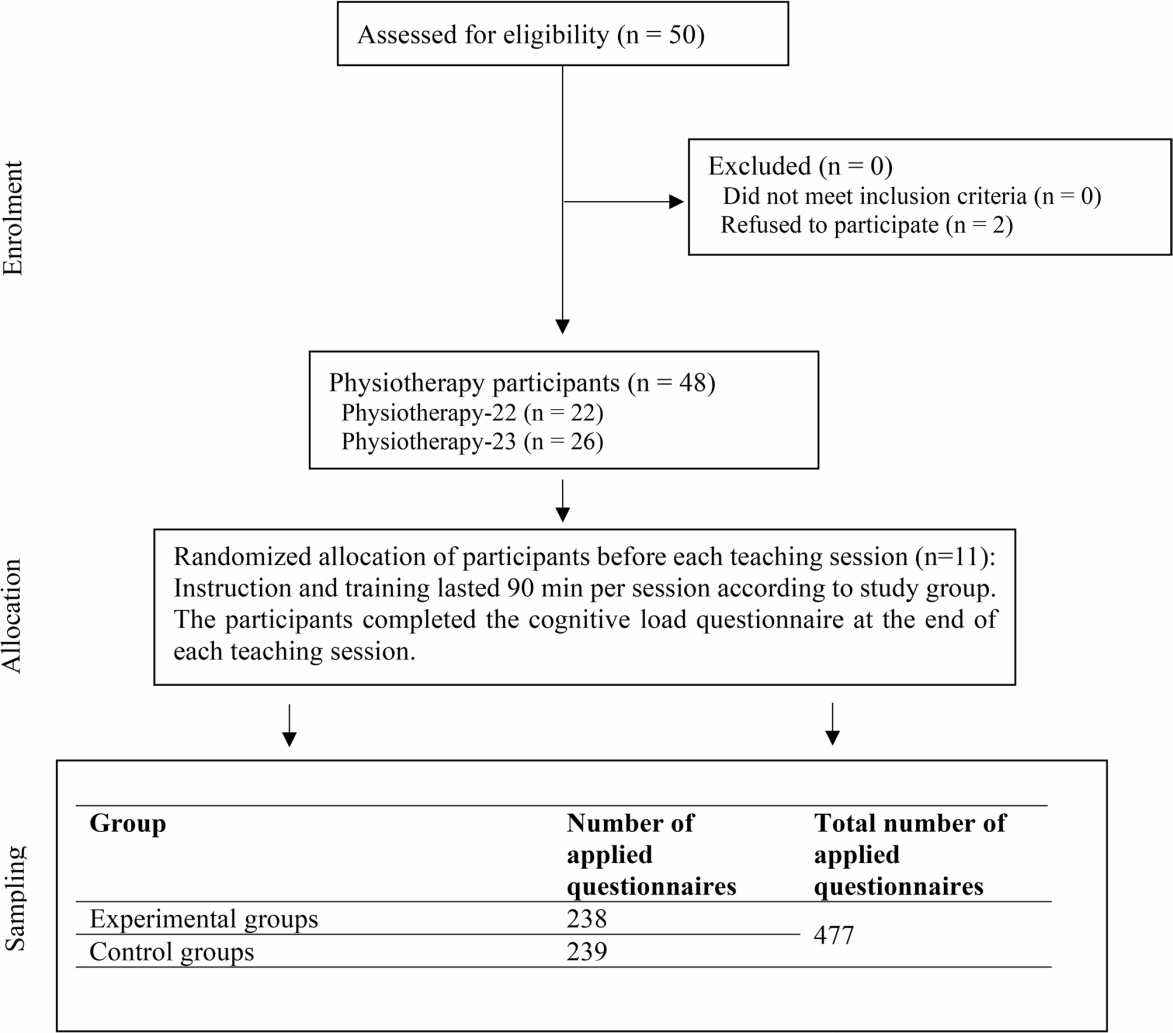
Flow of participants throughout the study course

The students were randomized at the beginning of each of the 11 manual therapy sessions and filled out the questionnaire at the end of each session. 239 questionnaires were completed in the series practice group and 238 in the individual practice group, resulting in a total of 477 questionnaires. Individual students missed 51 sessions due to illness, so no questionnaires could be collected in these cases. The flow of participants throughout the study is shown in Fig. 1.

### Primary outcome: global cognitive load

**Fig. 2:**
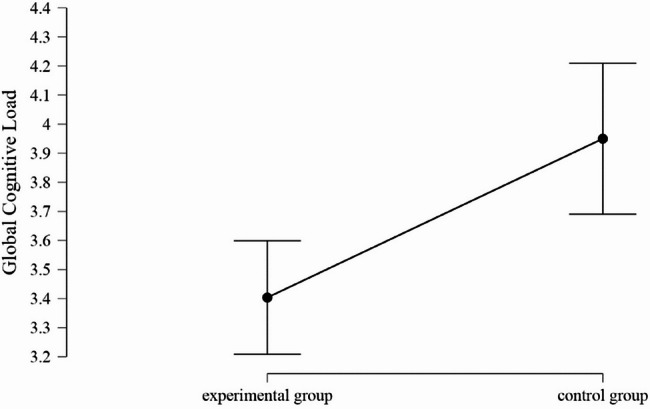
Means and 95% confidence intervals of the global cognitive load in the series practice group and the individual practice group (assessed on a 9-point Likert scale)

As shown in Figure 2, the global cognitive load measured on a 9-point-scale was significantly lower in the individual practice group than in the series practice group, MD = −0.55 (95% CI −0.87 to −0.22).

### Secondary outcome: components of cognitive load

**Fig. 3:**
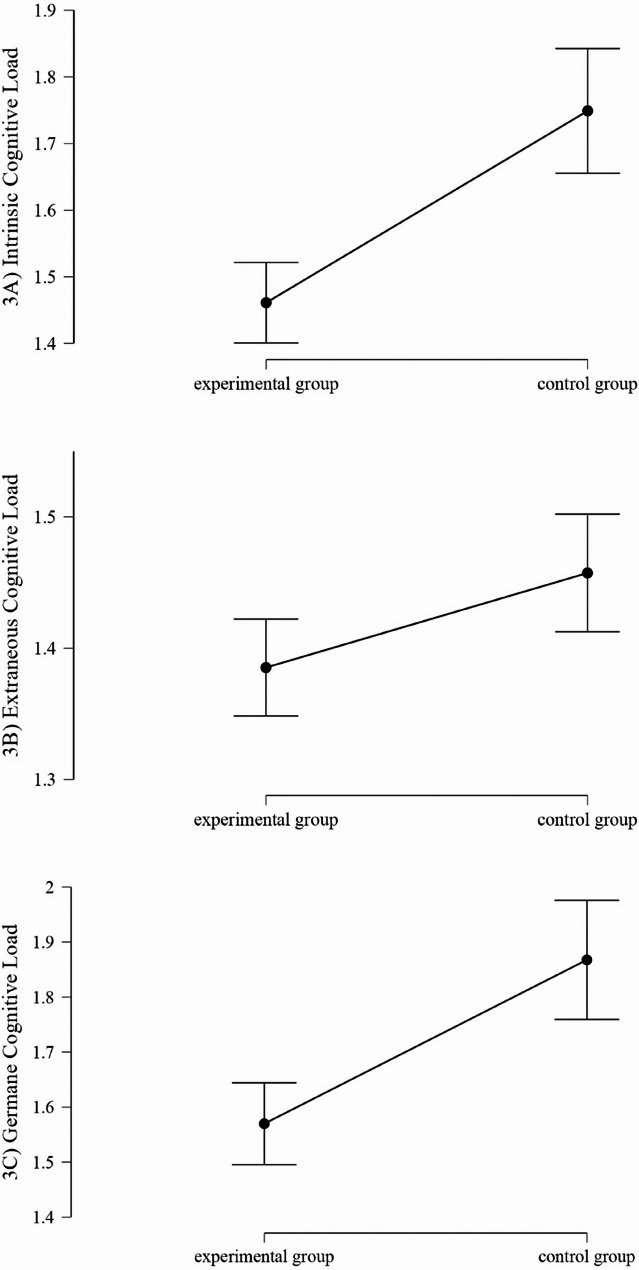
Means and 95% confidence intervals of the cognitive load categories in the series practice group and the individual practice group (assessed on a 4-point Likert scale)

The internal consistency (Cronbach’s α) for the items of each cognitive load category was measured as follows: intrinsic cognitive load: 0.83, extraneous cognitive load: 0.69, and germane cognitive load: 0.88. The means of all three cognitive load categories are depicted in Fig. 3a for the series and individual practice groups, respectively.

The intrinsic cognitive load was significantly lower in the individual practice group than in the series practice group, MD=−0.29 (95% CI −0.40 to −0.18). The participants in the individual group found it easier to carry out the treatment technique in the prescribed manner, identify the normal anatomy and landmarks, and keep track of the manual therapy findings than the participants in the series-group did.

In the extraneous cognitive load category, there was a slight difference between the individual practice group and the series practice group, MD=−0.07 (95% CI −0.13 to −0.01). The supervisor’s instructions were associated with slightly less cognitive load for the individual group than for the series practice group, MD=−0.1 (95% CI −0.02-0.00). Additionally, the manner of the supervisor's instructions was associated with a lower cognitive load for the individual group than for the series practice group, MD = −0.1 (95% CI −0.02 to −0.00). The participants in both groups felt equally distracted by the presence of other people in the room, were equally distracted by the environment, and were equally distracted by unrelated thoughts during the treatment.

The germane cognitive load category was also significantly lower in the individual practice group than in the series practice group, MD=−0.29 (95% CI −0.43 to −0.17). The participants in the individual group made less mental effort to learn how to carry out the new manual therapy technique, to identify or understand the anatomy of the particular treated structure, and overall, they had to make less mental effort to learn the new manual therapy treatment technique.

## Discussion

Learning about manual therapy plays a central role in professional physiotherapy practice and patient care, which involves managing pain and improving the function and movement of the musculoskeletal system. In this study, we utilized cognitive load theory to compare two approaches to the introduction and practice of manual therapy techniques by physiotherapy students. The following section discusses the results of this study and their interpretation, implications and limitations in the context of the reported literature.

Compared with students who were taught a series of 3–4 techniques consecutively, students who received individual instruction and practice in manual therapy techniques reported lower global cognitive loads as well as lower intrinsic, extrinsic, and germane cognitive loads.

These findings support our hypothesis that teaching and practising of manual therapy techniques individually may reduce the complexity and interactivity of the information that students must process by breaking down the learning content into smaller, more manageable units [15, 19, 28, 29].

The physiotherapy students in this study may have been able to better focus their attention on practising the specific details and nuances of one technique without the cognitive burden of switching between different techniques. With this approach, students may have been able to focus their cognitive resources on constructing and refining schemas for each specific technique, giving them a better opportunity to practice and automate their skills more effectively and to allocate their cognitive resources more efficiently to understanding and performing the individual technique [30]. In contrast, the teaching and practice of the techniques in a series may have led to students experiencing cognitive overlap, in which the elements of one technique interfere with the recall and application of another.

The differences in extraneous cognitive load among physiotherapy students were found to be less pronounced than those of other types, which may be attributable to the heterogeneity of the items. The items in this category assessed various factors, including the manner of the instructor’s directions, distractions from other students in the room, environmental distractions, and distractions from one’s own thoughts. The results indicate that only the teaching instructions in the individual teaching a practice group were associated with a slightly lower cognitive load.

This study has several implications for curriculum design and teaching approaches for the teaching and practice of manual therapy techniques for physiotherapy students. Educators might consider structuring their curriculum to teach and practice manual therapy techniques individually rather than in series. This approach can be integrated into both the theoretical and the practical components of a curriculum. Teachers may adopt a more segmented teaching strategy, in which each technique is given focused teaching time [31]. This could include separate sessions for each technique with ample time for practice and more targeted and specific feedback before moving on to the next technique [32]. The use of incremental learning and scaffolding techniques could further support the reduction of intrinsic cognitive load [33]. Additionally, by gradually increasing the complexity of tasks and providing appropriate support at each stage, educators can help students build their skills and knowledge in a more structured and manageable manner [6, 13, 34, 35]. This should also be reflected in supportive learning materials, such as step-by-step guides to reinforce techniques via a technique learning approach [36, 37].

This study has limitations that should be considered in the interpretation of its results. First, this was a single-centre study. Future studies should examine transferability to other organizational and educational contexts. The teacher of the training sessions for manual therapy was also the researcher of this study, which may have led to unintentional and unrecognized bias. A standardized teaching script was applied to reduce this potential bias. Additionally, although no manual therapy techniques were systematically taught prior to the start of the manual therapy course, it cannot be ruled out that students either acquired manual therapy techniques independently or had already practiced these techniques during their physiotherapy internships. It also cannot be ruled out that more motivated students may have practiced more independently than less motivated students, which could have influenced the results. Furthermore, it must be considered that the repeated completion of the questionnaire may have led to a certain familiarity with the answers and thus to a rather superficial completion of the questionnaire. Additionally, students were not assigned a personal ID, excluding the possibility to conduct repeated measure analyses.

In this study, a 4-point Likert scale was used to measure the categories of cognitive load instead of the 11-point Likert scale previously reported by Sewell and colleagues [27]. The use of this larger scale may have led to more nuanced measurement results. Also, this study primarily relies on Sweller’s cognitive load theory as a framework for working memory overload and thus a limitation on the transfer of acquired knowledge into long-term memory. However, there are other theories of frameworks for how skills can be acquired. For example, the Ecological Dynamic Framework, an innovative approach, emphasizes the interconnectedness of performer, environment, and task as a central point for understanding the development of movement patterns [38]. The generalizability of the results of this study to other instruments for measuring cognitive load needs to be examined. Further research is needed to refine measurement tools, particularly for extraneous cognitive load, and to explore possible short- and long-term effects of individualized instruction on skill retention and clinical performance.

## Conclusions

This randomized study of physiotherapy students revealed that teaching and practising one manual technique at a time may reduce the students’ cognitive load compared with teaching and practising them in a series of 3–4. The findings could have both theoretical and practical significance, as they highlight the potential for educators to apply empirical insights from cognitive load theory to optimize instructional strategies, thereby potentially enhancing learning efficiency.

## Supplementary Information

Appendice.docx. 1. Overview of manual therapy techniques for each joint.

## Data Availability

The data that support the findings of this study are available from Mr. René Gärtner, but restrictions apply to the availability of these data, which were used under license for the current study, and so are not publicly available. Data are however available from the authors upon reasonable request.
