# Supplementary material for: Teaching a single manual therapy technique at a time reduces cognitive load in physiotherapy students: a randomized controlled educational study
Source: BMC Med Educ. 2025 Oct 15;25:1422. doi: 10.1186/s12909-025-08083-w (PMC12523193; doi:10.1186/s12909-025-08083-w)
Supplement: Supplementary file 1 — Appendice.docx. 1. Overview of manual therapy techniques for each joint. [file 12909_2025_8083_MOESM1_ESM.docx]

Overview of manual therapy techniques for each joint

| Region/Joint | Name of the Manual Therapy technique |
| --- | --- |
| **Session 1** | |
| **Shoulder Girdle** | |
| Sternoclavicular joint  Acromioclavicular joint | 1. Joint-play for elevation in a caudo-lateral direction 2. Traction for elevation in a caudo-lateral direction 3. Joint-play for elevation in a caudal-medial direction 4. Traction for elevation in a caudal-medial direction |
| **Upper Extremity** | |
| Shoulder joint | 1. Translatory gliding in a caudo-lateral direction 2. Translatory gliding in a dorso-lateral direction 3. Translatory gliding in an antero-medial direction |
| **Session 2** | |
| Elbow joint | 1. Traction joint-play 2. Translatory gliding in a medial direction 3. Translatory gliding in a lateral direction |
| Proximal & Distal Radio-Ulnar joint (PRUG & DRUG) | 1. Translation in the PRUG for supination restriction 2. Translation in the PRUG for pronation restriction 3. Translation in the DRUG for supination restriction 4. Translation in the DRUG for pronation restriction |
| **Session 3** | |
| Wrist joint  Carpal joints  Finger joints (MCPs, PIPs, DIPs etc.) | 1. Translatory gliding in a dorsal - palmar direction 2. Translatory gliding in an ulnar – radial direction 3. Translatory gliding of the respective carpal bones against   each other   1. Traction and translatory gliding in all possible directions |
| **Session 4** | |
| **Lower Extremity** | |
| Hip joint 1 | 1. Traction from a supine position with extended knee joint 2. Traction from a supine position with flexed knee joint 3. Traction from a prone position with extended knee joint |
| Hip joint 2 | 1. Curved gliding (roll-gliding) in a supine position 2. Curved gliding (roll-gliding) in a side-lying position 3. Translatory gliding in a ventral direction in a prone position 4. Translatory gliding in a ventral-lateral direction in a prone position |
| **Session 5** | |
| Knee joint  Tibio-Fibular joint | 1. Translatory gliding in a caudal direction in 90° knee flexion 2. Translatory gliding in a cranial direction in 90° knee flexion 3. Translatory gliding in a dorsal-ventral direction |
| **Session 6** | |
| Upper ankle joint  Lower ankle joint | 1. Translatory gliding in a dorsal direction 2. Translatory gliding in a ventral direction 3. Translatory gliding in supination and pronation |
| **Session 7** | |
| Tarsal joints  Big toe joint (Art. meta-tarsophalangeal I)  Toe joints (MTPs, PIPs, DIPs etc.) | 1. Translatory gliding of the respective tarsal bones against each other 2. Traction and translatory gliding in the possible directions 3. Traction and translatory gliding in the possible directions |
| **Session 8** | |
| **Spine** | |
| Cervical Spine | 1. Gliding in an anterior-posterior direction of the entire cervical spine 2. Gliding in a lateral direction of the entire cervical spine 3. Traction treatment of the entire cervical spine 4. Gliding mobilization treatment of the individual vertebral bodies in an anterior-posterior direction |
| **Session 9** | |
| Cervicothoracic junction (CTJ) | 1. Segmental extension of the CTJ 2. Segmental flexion of the CTJ 3. Nonspecific traction of the CTJ |
| Thoracic Spine | 1. Butterfly technique 2. Gliding mobilization treatment of the vertebral bodies in an anterior-posterior direction 3. Traction treatment of the vertebral bodies 4. Traction treatment of the vertebral bodies with rotation |
| **Session 10** | |
| Lumbar Spine | - 1. Distraction   2. Rotation technique: pelvis anterior   3. Rotation technique: pelvis posterior   4. Gliding mobilization treatment of the vertebral bodies in an anterior-posterior direction |
| **Session 11** | |
| Ilio-Sacral joint (ISj) | 1. ISj gapping 2. Superior iliac spine “up” 3. Superior iliac spine “down” |
